# Knowing When to Self-Eat – Fine-Tuning Autophagy Through ATG8 Iso-forms in Plants

**DOI:** 10.3389/fpls.2020.579875

**Published:** 2020-11-03

**Authors:** Svetlana Boycheva Woltering, Erika Isono

**Affiliations:** ^1^Department of Biology, University of Konstanz, Konstanz, Germany; ^2^Zukunftskolleg, University of Konstanz, Konstanz, Germany

**Keywords:** plant autophagy, adaptation mechanism, recycling, abiotic stress, regulator, regulation target

## Abstract

Autophagy is a catabolic process that takes place under both normal and adverse conditions and is important for the degradation of various organelles and proteins that are no longer needed. Thus, it can be viewed as both a constitutive recycling machinery and an adaptation mechanism. Increase in the activity of autophagy can be caused by multiple biotic and abiotic stress factors. Though intensive research in the past decade has elucidated many molecular details of plant autophagy, the mechanisms of induction and regulation of the process remain understudied. Here, we discuss the role of ATG8 proteins in autophagic signaling and regulation with an emphasis on the significance of ATG8 diversification for adapting autophagy to the changing needs of plants.

## Introduction

Plants are unable to escape from unfavorable environmental conditions or damaging interactions with other organisms. Hence, in order to survive, they need to adapt to the changes in their surroundings as fast as possible. Due to these characteristics, it is important for plants to efficiently acquire essential elements, produce and reuse metabolites, and optimize energy consumption by recycling of cellular components.

One mechanism to degrade and recycle cytoplasmic material and provide building blocks in eukaryotic cells is autophagy (Li and Vierstra, 2012; Liu and Bassham, 2012; Avin-Wittenberg et al., 2018). Microautophagy occurs through invagination of the tonoplast engulfing cytoplasmic material in autophagic bodies while macroautophagy, hereafter referred to as autophagy, involves the formation of a cup-shaped phagophore which closes to form the autophagosome. Research conducted in the yeast *Saccharomyces cerevisiae* identified the main players in autophagy and revealed the detailed molecular mechanisms of autophagic degradation (Tsukada and Ohsumi, 1993; Noda et al., 1995; Ohsumi, 2001). Further studies with model organisms, including *Arabidopsis thaliana*, demonstrated high degree of conservation for the autophagic proteins in plants (Liu and Bassham, 2012).

**FIGURE 1:**
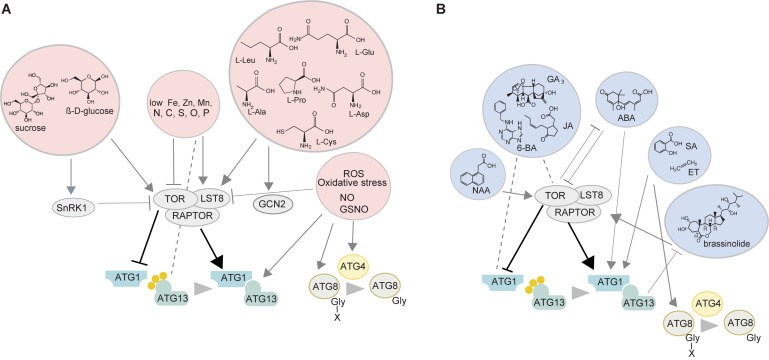
Autophagy sensors and regulators with or without the involvement of the TOR kinase. **(A)** Sugars, mineral nutrients, amino acids, and ROS affect autophagy. Glucose activates TOR while the lack of it inhibits the TOR kinase thus activating autophagy. Furthermore, SnRK1 kinase can be involved in sugar sensing upstream of TOR. Low availability of nutrients activates autophagy with C and N starvation acting through TOR inhibition. P-deficiency stimulates autophagy independently of TOR, although LST8 was shown to act as an intermediate. Lack of Fe, Zn, and Mn also activates autophagy but the participation of TOR is not clear. Changes in the levels of branched chain amino acids, with or without the involvement of TOR have been reported to modulate TOR and autophagic activity in mammals. However, the only well-studied amino acid in plants in this aspect is cysteine where the levels of the sulfur precursor affect the activity of the TOR kinase itself, while the C/N precursor levels are perceived by GCN2 kinase. ROS can activate autophagy via TOR inhibition or directly influencing multiple ATG genes/proteins downstream of the TOR kinase. **(B)** Phytohormones modulate the activity of TOR and/or autophagy. Auxin (NAA) stimulates TOR thereby inhibiting the autophagy process. Brassinosteroids are also able to inhibit autophagy through TOR activation while autophagy itself can affect BR signaling by degrading components of it. ABA is involved in an interplay with the TOR kinase by inhibiting its activity under stress, thereby promoting autophagy, while under favorable conditions TOR suppresses ABA signaling. A direct connection between ABA and autophagy is indicated by the involvement of two ATG8-interacting proteins ATI1 and ATI2 in ABA-mediated germination and salt tolerance. The presence of less GA in autophagy mutants indicates its possible involvement in autophagy regulation although the exact mechanisms have not been established. Ethylene stimulates autophagy by directly activating ATG8 homologs or via ROS. The exact relationships of JA and CK with TOR and autophagy have not been established. SA stimulates autophagy involved in senescence and plant immunity possibly with the involvement of ROS. NO can regulate hypoxia responses by S-nitrosylating various proteins including the master regulator of NO signaling *S*-nitrosoglutathione reductase thus exposing its ATG8-interacting motif and targeting it for degradation. TOR, target of rapamycin; RAPTOR, regulatory-associated protein of mTOR; LST8, lethal with sec 13 8; SnRK1, sucrose non-fermenting related kinase 1; GCN2, general control non-depressible 2; ROS, reactive oxygen species; ATG, autophagy-related; NAA, naphthaleneacetic acid; 6-BA, 6-benzyladenine; GA3, gibberellic acid; JA, jasmonic acid; ABA, abscisic acid; SA, salicylic acid; ET, ethylene; BR, brassinolide; NO, nitric oxide.

The induction of autophagy in plants involves several protein kinases (Figures 1A,B). The best studied example is the target of rapamycin (TOR) kinase which is a negative regulator of autophagy and its downregulation or inhibition leads to constitutive activation of autophagy (Liu and Bassham, 2010). TOR belongs to the TORC1 complex together with its target recognition cofactor REGULATORY-ASSOCIATED PROTEIN OF mTOR (RAPTOR), and the stabilizer LETHAL WITH SEC13 8 (LST8) (Shi et al., 2018). Once activated, the TOR kinase transfers the signals downstream by phosphorylating the ATG1/ATG13 complex thus stimulating autophagic vesiculation that involves the decoration of the phagophore with phosphatidylinositol-3 phosphate (PI3P) and the delivery of lipids to the expanding phagophore (Suttangkakul et al., 2011). The ubiquitin-like ATG8 is then processed by the cysteine protease ATG4, which leads to the exposure of the c-terminal glycine of ATG8, allowing its recruitment to the phagophore through the attachment of the phospholipid phosphatidylethanolamine (PE) (Kirisako et al., 2000). Following closure, the newly formed double membrane delimited autophagosome is transported to the vacuole and fuses with the tonoplast, subsequently releasing its single-membrane content as an autophagic body into the vacuole.

Autophagy in plants was initially described as a system for bulk recycling of cytoplasmic material (Bassham, 2009). However, it is becoming increasingly evident that the process can be highly selective and requires strict regulation on multiple levels. An enormous body of studies of autophagy in plants elucidated the functions of core components and has been discussed in multiple excellent review articles (Ohsumi, 2001; Marshall and Vierstra, 2018; Shi et al., 2018; Yoshimoto and Ohsumi, 2018). Although some aspects of autophagy modulation have been well-studied, the detailed mechanisms remain elusive. In this review, we focus on regulation of autophagy mainly through ATG8 diversification and specialization.

## Tor Kinase as a Central Modulator of ATG Proteins

Small molecules, mineral nutrients, reactive oxygen species (ROS) and phytohormones can influence the activity of autophagy and ATG proteins in plants in a TOR-dependent manner. Amino acid availability and abundance of their degradation products affect metabolic and developmental processes (Hildebrandt et al., 2015) including autophagy (Meijer et al., 2015). However, only cysteine has been described in detail in plants, with sulfur availability being sensed by the TOR kinase and the carbon/nitrogen (C/N) precursors by GENERAL CONTROL NON-DEPRESSIBLE2 (GCN2) kinase (Dong et al., 2017) (Figure 1A). High levels of sulfur lead to increased glucose levels, and subsequently activate TOR, whereas reduced glucose levels during sulfur deficiency or impaired photosynthesis inhibit TOR and activate autophagy (reviewed in Fu et al., 2020). Glucose is the likely transmitter of the signal between sulfur and TOR (Dong et al., 2017) but whether it could convey the availability of other nutrients in the context of autophagy is unclear. The mechanisms of GCN2 perception in plants are unknown, although GCN2 activation by uncharged tRNAs appears to be universal in eukaryotes (Li et al., 2013) (Figure 1A). Prolonged carbon starvation (Huang et al., 2019) and phosphate deficit which causes ER stress (Naumann et al., 2019) induce autophagy independently of TOR, although recent research in Chlamydomonas suggests LST8, the stabilizer of TOR, is involved in phosphate starvation response (Couso et al., 2020).

The connection between autophagy and nitrogen metabolism, transport, and remobilization has been explored by the Masclaux-Daubresse lab and is covered in recent reviews (Masclaux-Daubresse et al., 2017; Chen et al., 2018). We will only mention that N depletion strongly inhibits TOR and activates autophagy, although its deficit also leads to accumulation of sugars, including glucose, which activates TOR and inhibits autophagy (Cao et al., 2019). Zinc deficit has also been shown to induce autophagy in *Arabidopsis thaliana* although the mechanisms are unclear (Shinozaki et al., 2020). In mammalian cells, iron is released from storage by Nuclear Receptor Coactivator4 (NCOA4) autophagy receptor mediated ferritin degradation in the lysosome (ferritinophagy) (Mancias et al., 2014; Mancias et al., 2015). In plants, multiple autophagy mutants of Arabidopsis have impaired translocation of iron, zinc and manganese, to the seeds, suggesting that deficit of these elements could induce autophagy (Pottier et al., 2018) (Figure 1A).

Phytohormones have been shown to regulate the TOR kinase and thus have the potential to affect TOR-dependent autophagy (reviewed in Fu et al., 2020). For example, auxin inhibits stress-induced autophagy by stimulating TOR activity (Schepetilnikov et al., 2017) (Figure 1B) or blocking autophagosome formation under certain stresses (Pu et al., 2017). Brassinosteroids (BRs) use the TOR kinase to inhibit autophagy and enhance their signaling through brassinazole-resistant (BZR)1 transcription factor at the same time (Zhang et al., 2016) (Figure 1B). BZR1 itself was shown to activate the transcription of the autophagy adaptor *Neighbour of BRCA (NBR)1*, promoting its own selective degradation (Chi et al., 2020). Direct involvement of gibberellins (GA) in the regulation of autophagy is unclear although lower levels of GA were detected in the rice autophagy mutant *osatg7-1* (Kurusu et al., 2017). The stress hormone abscisic acid (ABA) represses TOR activity (Wang et al., 2018), leading to autophagy activation while TOR itself inhibits ABA signaling under favorable environmental conditions. High levels of salicylic acid (SA) have been associated with autophagy-mediated senescence and programmed cell death (PCD) (Yoshimoto et al., 2009). In Arabidopsis, SA also accumulates during flooding increasing ROS levels, and stimulating autophagy (Chen et al., 2015). Both SA and PCD are strongly associated with pathogen response and plant immunity (Liu et al., 2016; Leary et al., 2019). Autophagy regulation in the context of plant immunity is extensively covered in other recent reviews (Üstün et al., 2017; Leary et al., 2019).

## Fine-Tuning Autophagy Through ATG8

One of the proteins central to the autophagic process, the ubiquitin-like ATG8, usually requires activation through post-translational cleavage by the ATG4 protease at the c-terminus. This process seems to be controlled for instance by ROS, that were shown to activate ATG4 in Chlamydomonas (Pérez-Pérez et al., 2012, 2016). Both ATG8 and ATG4 have more than one homolog in most plant species and several ATG4 homologs have been shown to interact preferentially with distinct ATG8 iso-forms (Woo et al., 2014; Seo et al., 2016). These observations imply that ATG8s and ATG4s could contribute to fine-tune specific and efficient induction of autophagy.

ATG8 can interact with receptor and adaptor proteins containing either ATG8-interacting motif [AIM/LIR; LIR-docking site (LDS)] or Ubiquitin-interacting motif [UIM; UIM-docking site (UDS)] (Marshall et al., 2019). UDS-interacting adaptors are the proteasome degradation subunit Regulatory particle non-ATPase (RPN)10 and the proteins from the Plant Ubiquitin Regulatory X Domain (PUX) family, which have diverse cellular functions (Marshall et al., 2015; Wen and Klionsky, 2016). LDS-interacting are NBR1 and the ATI (ATG8-interacting protein)1 and ATI2 proteins targeting plastid proteins for degradation upon carbon starvation (Honig et al., 2012; Michaeli et al., 2014). Known autophagy receptors are: the Arabidopsis Orosomucoid (ORM) proteins 1 and 2, involved in Flagellin-Sensing 2 (FLS2) receptor kinase degradation (Yang et al., 2019); DSK2, targeting the brassenosteroid pathway regulator BES1 for degradation (Nolan et al., 2017); the Arabidopsis TSPO protein regulating the levels of PIP2;7 aquaporin on the cell surface (Hachez et al., 2014). Most recently, a dehydrin in *Medicago truncatula* MtCAS31 was found to promote autophagy under drought stress through interaction with ATG8 (Li et al., 2019). Autophagy in the context of hypoxia responses can be regulated by nitric oxide (NO) which can *S*-nitrosylate various proteins including the master regulator of NO signaling *S*-nitrosoglutathione reductase, thus exposing its ATG8-interacting motif and targeting it for degradation (Zhan et al., 2018). It is unclear whether the highly variable AIM/LIR provides another layer of specificity in terms of different ATG8 iso-forms interacting with different variants of the motif.

Given the diversity of ATG8s in higher plants, it is difficult to conclude on the importance of each iso-form based on single knockout mutants due to functional redundancy. Different ATG8s appear to be induced by varying stress factors or to alleviate them although some overlap has been discovered (Vanhee et al., 2011; Xia et al., 2012; Luo et al., 2017; Chen et al., 2018; Li et al., 2019). The ability of different ATG8 iso-forms to interact with different effectors implies the existence of unique functions of the ATG8 homologs. Unfortunately, all the functional data so far have been obtained from a handful of model organisms and is not equally covering all the homologs. While it is essential that more *in vivo* and *in planta* data are acquired, in the next section we use existing resources such as available plant genomes together with transcriptomics and proteomics data to explore ATG8 specificities in the plant kingdom.

## Significance of the ATG8 Diversity

**FIGURE 2:**
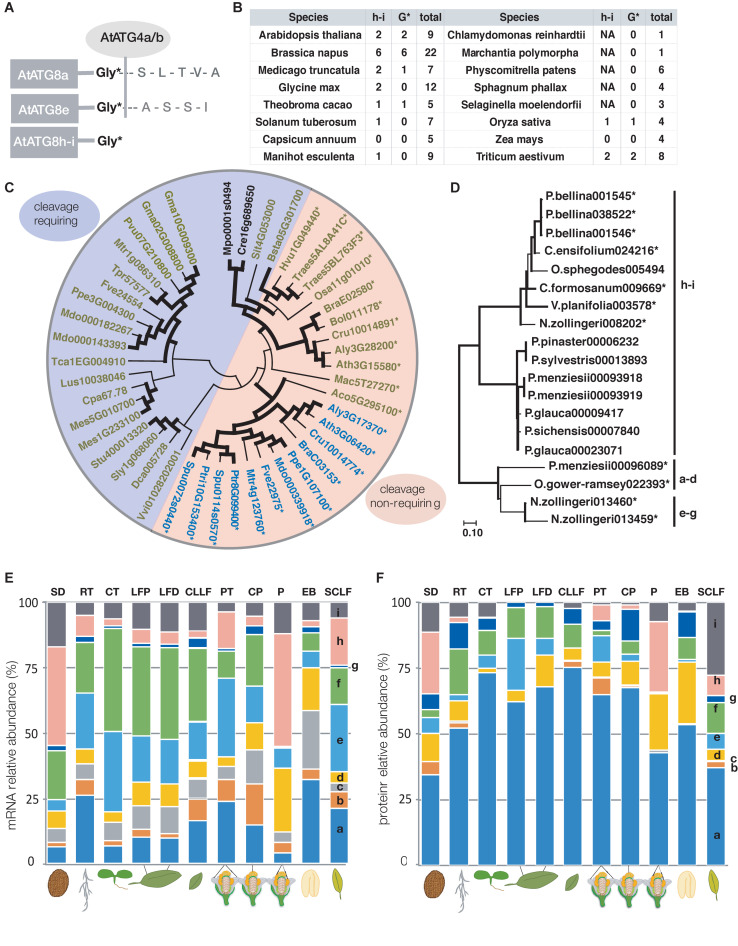
ATG8 analysis. **(A)** Schematic representation of cleavable and non-cleavable ATG8 iso-forms in Arabidopsis. **(B)** The table lists the homologs in the ATG8h-i group for a given species, the total number and the number of homologs with an exposed glycine. In some cases, the ATG8h-i group members have been secondarily lost such as in *C. annuum* and *Z. mays*. **(C,D)** Phylogenetic tree of ATG8h-i homologs of selected mono- and eudicot species and gymnosperms and orchids, respectively. Protein coding sequences were taken from Phytozome 12 (Goodstein et al., 2012) in **(C)** and from Gymno Plaza 1.0 (Proost et al., 2009) and Orchidstra 2.0 (Chao et al., 2017) for **(D)**. **(C)** Unicellular algae have a single ATG8, while mosses can have one or multiple homologs both groups clustering together and completely separately from the ATG8h-i group of homologs of the higher plants. Some homologs of the ATG8h-i group have an exposed catalytic glycine (marked with an asterisk) such as all the sequenced *Brassicaceae* species. ATG8h-i members with and without an exposed glycine could be in the same species. The ATG8h-homologs are shown in blue and the ATG8i-homologs are depicted in green. The algae/mosses group is shown in black. In **(C)**, all homologs with an exposed catalytic glycine are marked with an asterisk and the entire segment of the tree is colored in orange and the cleavable iso-forms are colored in blue. **(D)** The clades are indicated next to the tree, while the homologs with an exposed G are marked with an asterisk. The Evolutionary analysis was conducted using the Maximum Likelihood method and General Time Reversible model (Nei and Kumar, 2000) for both **(C,D)**. The bootstrap consensus tree inferred from 1000 replicates is taken to represent the evolutionary history of the taxa analyzed (Felsenstein, 1985). Branches corresponding to partitions reproduced in less than 50% bootstrap replicates are collapsed. The percentage of replicate trees in which the associated taxa clustered together in the bootstrap test (1000 replicates) are shown next to the branches and the ones equal to or above 60% are indicated by a bold black line (Felsenstein, 1985). Initial trees for the heuristic search were obtained automatically by applying Neighbor-Join and BioNJ algorithms to a matrix of pairwise distances estimated using the maximum composite likelihood (MCL) approach, and then selecting the topology with superior log likelihood value. A discrete Gamma distribution was used to model evolutionary rate differences among sites [5 categories (+ G, parameter = 0.9115)]. The rate variation model allowed for some sites to be evolutionarily invariable ([ + I], 15.22% sites). This analysis involved 45 and 19 protein coding nucleotide sequences with 335 and 309 positions in the final dataset for **(C)** and **(D)** respectively. Evolutionary analyses were conducted in MEGA X (Kumar et al., 2018; Stecher et al., 2020). **(E,F)** Relative abundance of ATG8 transcript and protein, respectively, based on a dataset from Mergner et al. (2020) (data file 41568_2020_2094_MOESM4) containing experimental data for 30 different tissues and in both **(E)** and **(F)** non-transformed values were used for generating the graphical representation. Transcript abundance was estimated using TPM (transcript per million) and protein abundance estimation was based on iBAQ (intensity-based absolute quantification). ATG8 protein iso-forms a, d, and f can be found in every tissue examined. Values are for selected organs, tissues, and developmental stages: SD – dry seed; RT – root; CT – cotyledon; LFP – rosette leaf 7 proximal part; LFD – rosette leaf 7 distal part of the leaf; CLLF – cauline leaf 1; PT – petal; CP – carpel; P – pollen; EB – embryo; SCLF – senescent leaf.

ATG8 proteins have shown a tendency toward increase in number of iso-forms and diversification in multicellular organisms. Yeast and algae have a single homolog while in the genomes of higher plants, multiple homologs can be found (Kellner et al., 2017). The nine iso-forms (ATG8a-i) of Arabidopsis have been investigated more in depth revealing that they belong to three separate groups – a-d, e-g, and h-i (Seo et al., 2016; Kellner et al., 2017). A characteristic of many members of the ATG8h-i group is that they have an exposed catalytic glycine residue, and do not require cleavage by the ATG4 protease for activation (Figure 2A) (Seo et al., 2016; Kellner et al., 2017).

Analyzing several model and crop species showed that the presence of ATG8h-i group members is not strictly associated with the total number of ATG8 homologs in each species (Figure 2B). For example, in maize (*Zea mays*), none of the four ATG8s belong to the ATG8h-i group, while one of the four rice (*Oryza sativa*) homologs is cleavage-free. Additionally, while Medicago (*Medicago truncatula*) and soybean (*Glycine max*) have two ATG8h-i iso-forms each, only one in Medicago is cleavage-free. The rapeseed (*Brassica napus*) has six h-i homologs, all ending with a glycine (G) and as the plant is a recent alopolyploid (Lu et al., 2019), it could serve as a model to study ATG8 diversification and mechanisms of retention. While *Brassicaceae* species have only ATG8h-i iso-forms with an exposed glycine, some ATG8i-homologs from other taxonomic groups have additional amino acids at the C-terminus. The cleavage-free and cleavage-requiring forms separate from each other as shown by Maximum likelihood analysis (Figure 2C). It is yet to be established whether this exposed glycine residue provides any advantage to the ATG8 protein that possesses it. If cleavage free iso-forms are constitutively active, they might be important for fast induction of autophagy under stress conditions. However, involvement in a developmental process cannot be excluded based on the current understanding of the iso-forms which prompted further analyses of the ATG8 family. Exploring several gymnosperm genomes indicated, that while acquisition of multiple ATG8 homologs can be associated with multicellularity, the non-cleavable forms appeared together with seeds. Interestingly, the gymnosperms homologs with an exposed G belong to the a-d group (Figure 2D) and the limited number makes it difficult to conclude on their significance. Analyzing additional mono- and dicots such as the primitive *Amborella trichopoda* (*Amborellaceae* family) (Endress and Igersheim, 2000) and members of the *Orchidaceae* family, showed that cleavage free ATG8s exist in each case and with only two exceptions belong to the h-i group (Figure 2D). Whether the mass appearance of cleavage free ATG8s can be associated with any of the characteristics new to seed plants remains to be investigated. It is however established, that autophagy plays an important role in male reproduction in plants (reviewed in Norizuki et al., 2020) and in rice, endosperm development (Sera et al., 2019). However, the current advances in ATG8s do not allow conclusions on specific functions of the homologs to be made. A step in the right direction would be investigating the possible unique roles of ATG8 iso-forms.

## Expression and Abundance of ATG8S

In order to estimate the potential for diversity of function of the ATG8s, we next analyzed data from two recent large-scale studies dealing with transcriptional regulation and with proteomics, respectively, conducted in Arabidopsis. The first study identified 225 transcription factors (TFs) by yeast one-hybrid screen able to bind the promoters of four *ATG8* genes, namely *ATG8a*, *b*, *e*, and *h* (Wang et al., 2019) and thus having the potential to activate their transcription. Surprisingly, only 19 of those TFs were shared by all four genes. The *ATG8e* promoter interacted with 71 unique TFs. Transcriptional activation by phytohormones such as ET can affect the expression of various *ATG* genes which makes it essential for survival during submergence in Arabidopsis (Chen et al., 2015; Hartman et al., 2019), most likely mediating the switch from hypoxia-associated fermentation to degradation of amino acids and fatty acids (Okuda et al., 2011; Zhu et al., 2018) (Figure 1B). ET is also able to stimulate the expression of *ATG8* homologs specifically in autophagy-dependent pollen development in petunia (*Petunia hybrida*) likely through ROS signaling or direct binding to their promoters (Okuda et al., 2011; Zhu et al., 2018). It is however currently unknown whether ET has identical affinity toward all *ATG8* promoters.

Apart from the evidence for differential transcriptional regulation of the ATG8 homologs, differences in protein levels have recently been documented by large scale quantitative transcriptome and proteome analysis of 30 different tissues (Mergner et al., 2020). While all *ATG8* genes seem to be expressed in every analyzed tissue (Figure 2E), only AtATG8a, d, and f protein iso-forms are ubiquitously present (Figure 2F). Notably, in root, petal, carpel and senescing leaves all ATG8 proteins can be found and ATG8a appears to be the predominant iso-form in all tissues (Figure 2F) (Mergner et al., 2020). The AtATG8h protein is very strongly expressed in pollen and dry seed, reflecting high mRNA levels in the same tissues, but completely missing in rosette and cauline leaves. The other member of the clade, AtATG8i, also has the highest relative protein abundance in senescent leaves but is also strongly present in pollen and dry seed, and cotyledons also contain relatively high levels. One possible explanation for the discrepancies in transcript and protein abundance of the ATG8 could be different turnover rates of the proteins. It also remains to be established to what extent the exposed c-terminal glycine could offer an advantage in different autophagy inducing conditions and whether the h and i iso-forms would require some other type of activation.

## Discussion

The presence of multiple regulation targets providing grounds for activity modulation of autophagy could be considered as an indicator that such intricate regulatory system is in fact necessary. It is without a doubt, that understanding the molecules and signals affecting the autophagy machinery would contribute to the elucidation of autophagy induction and regulation.

One of the most upstream targets of autophagy modulation, the TOR kinase, has recently featured in a phosphoproteomic screen combined with targeted proteomics analysis of interacting proteins, in *Arabidopsis thaliana*, identifying potential upstream and downstream components of the TOR network (Van Leene et al., 2019) that could be investigated together with many non-protein molecules in relation to autophagy regulation.

Increase in number of iso-forms and diversification of ATG8 in plants along the course of evolution might reflect the need to accommodate the requirements of the increased complexity of flowering species. It is possible that iso-form specific or clade-specific ATG8 functions exist or that certain homologs are more abundant in autophagosomes under given conditions, in specific tissues and organs or during certain developmental stages. Additionally, exploring the specificity of the ATG8 interactions and the significance of the cleavage free iso-forms, also by obtaining more functional data could further clarify the diversity of the ATG8 homologs in plants and contribute to the understanding of autophagy induction and regulation. In this line of thought, clarifying the molecular mechanisms regulating ATG4 and ATG8 would provide more insight into the process of fine-tuning autophagy.

## Author Contributions

SBW provided the initial draft while both SBW and EI were involved in the further editing of the manuscript and the preparation of the figures. Both authors conceptualized the idea.

## Conflict of Interest

The authors declare that the research was conducted in the absence of any commercial or financial relationships that could be construed as a potential conflict of interest.
